# Intranasal administration of *Clostridium butyricum* and its derived extracellular vesicles alleviate LPS-induced acute lung injury

**DOI:** 10.1128/spectrum.02108-24

**Published:** 2024-10-29

**Authors:** Li Liu, Cihua Zheng, Zhenyang Xu, Zhuoya Wang, Yuchun Zhong, Zhidong He, Wenming Zhang, Yuanbing Zhang

**Affiliations:** 1 Clinical School of Medicine, Jiangxi University of Chinese Medicine, Nanchang, Jiangxi, China; 2 The Institute of Translational Medicine, The Second Affiliated Hospital of Nanchang University, Nanchang University, Nanchang, Jiangxi, China; 3 Department of Rehabilitation Medicine, The Second Affiliated Hospital of Nanchang University, Nanchang, Jiangxi, China; Children's National Hospital, George Washington University, Washington, DC, USA

**Keywords:** ALI, *Clostridium butyricum*, extracellular vesicles, pulmonary microbiota, LPS

## Abstract

Acute lung injury (ALI) is associated with high morbidity and mortality rates. However, its clinical treatment is limited. Currently, the treatment of lung diseases by regulating the lung microbiota has become a research hotspot. In this study, we investigated the protective effects of the intranasal administration of *Clostridium butyricum* and its derived extracellular vesicles (EVs) against lipopolysaccharide (LPS)-induced ALI. The results demonstrated that compared with the LPS group, the pre-treatment group with *C. butyricum* and its EVs reduced the expression of pro-inflammatory cytokines and alleviated the symptoms in ALI mice by inhibiting the TLR4/MyD88 signaling pathway. Moreover, *C. butyricum* and its derived EVs inhibited the expression of apoptosis-related proteins and increased the expression of lung barrier proteins. Additionally, the intervention of *C. butyricum* changed the composition of the pulmonary microbiota. At the species level, LPS significantly increased the relative abundance of *Acinetobacter johnsonii*, while *C. butyricum* reversed this effect. In conclusion, these data demonstrate that intranasal administration of *C. butyricum* and its EVs can prevent LPS-induced ALI by reducing inflammation, inhibiting apoptosis, and improving lung barrier function. Additionally, *C. butyricum* regulated the pulmonary microbiota of mice to alleviate LPS-induced ALI.

**IMPORTANCE:**

The disorder of pulmonary microbiota plays an important role in the progression of acute lung injury (ALI). However, very few studies have been conducted to treat ALI by modulating pulmonary microbiota. In this study, the diversity and composition of pulmonary microbiota were altered in lipopolysaccharide (LPS)-induced ALI mice, but the ecological balance of the pulmonary microbiota was restored by intranasal administration of *Clostridium butyricum*. Moreover, the study reported the mechanism of *C. butyricum* and its derived extracellular vesicles for the treatment of LPS-induced ALI. These results reveal the importance of pulmonary microbiota in ALI disease. It provides a new approach for the treatment of ALI with new-generation probiotics.

## INTRODUCTION

Acute lung injury (ALI) is an acute lung disease caused by infection, trauma, shock, aspiration, and other factors (1). It is characterized by damage to the alveolar epithelial barrier, accumulation of inflammatory cells, and pulmonary edema (2). ALI can cause acute respiratory distress syndrome in severe cases and is characterized by high morbidity and mortality (3). As ALI pathogenesis remains obscure, the current treatment of acute lung injury is limited and predominantly involves the use of hormonal drugs, such as dexamethasone (4). Although hormonal drugs have good anti-inflammatory effects, they may lead to serious adverse reactions, such as hyperglycemia, immunosuppression, and osteoporosis (5). Therefore, it is imperative to identify drugs that can safely and effectively alleviate ALI.

Currently, ALI pathogenesis is unclear but may be related to the release of inflammatory cytokines and destruction of the pulmonary microvascular barrier, both of which are closely related to the pulmonary microbiota (6). It has been reported that the diversity of lung microbiota in mice is negatively correlated with the levels of inflammatory cytokines [interleukin (IL)-1α and IL-4]. This indicates that lung microbiota affects the release of inflammatory cytokines (7). The pulmonary microbiota plays an important role in maintaining the lung mucosal barrier and regulating lung immune function (8). Research demonstrates that, compared with healthy rats, the ecological imbalance in ALI rats is characterized by a decrease in pulmonary microbiota diversity or changes in components (9). Therefore, it has become a research hotspot to intervene in ALI and other lung diseases by regulating the lung microbiota.

Symbiotic microorganisms exist in well-balanced lung tissues, and there are 10–100 bacteria in every 1,000 host cells in the lung tissue (10). The composition of normal lung microbiota is relatively stable and highly diverse. The interaction between the lung microbiota and its host has reached a symbiotic relationship. An imbalance of microbes may play a pathogenic role by upregulating inflammatory signals or interfering with the production of cytokines. An imbalance in the pulmonary microbiota seriously affects homeostasis of the body (11). Nasal instillation of hesperidin-loaded nanoparticles can treat ALI by reducing cellular inflammation (12). It has also been reported that Sangju Qingjie Decoction can treat chronic obstructive pulmonary disease rats by increasing the abundance and diversity of pulmonary microbiota and reducing inflammatory factors, such as IL-6, IL-8, and tumor necrosis factor (TNF)-α (13). Basic research has demonstrated that *Scutellaria baicalensis* water decoction can improve lung infection by regulating lung microbiota and reducing inflammatory factors (14). It is reported that heat-killed *Clostridium butyricum* can reduce inflammatory cytokines, improve autophagy, inhibit nuclear factor kappa-B (NF-κB)/nucleotide oligomerization domain-like receptor 3 inflammatory pathway, and improve airway inflammation in asthmatic mice (15). *C. butyricum* is a Gram-positive bacterium belonging to *Clostridiaceae* family and *Clostridium* genus. It has been reported that *C. butyricum* can improve endometritis by inhibiting the signal pathway mediated by toll-like receptor 4 (TLR4) and increasing the expression of tight junction proteins occludin and Zona occludens protein 1 (ZO-1) in the uterine tissue (16). Basic research has demonstrated that *C. butyricum* can increase the diversity of the gut microbiota, reduce the inflammatory response in intestinal tissues dependent on the TLR4/myeloid differentiation primary response gene (88) (MyD88) pathway, and improve inflammation caused by *Salmonella* (17). According to one report, the oral administration of *C. butyricum* can reduce airway inflammation and alleviate ovalbumin-induced asthma (18). Related research has reported that *C. butyricum* can improve respiratory syncytial virus-induced lung inflammation in mice by regulating intestinal microbe (19). Although several studies have revealed the protective effects of *C. butyricum* in the human body, further research is required on the interactions between *C. butyricum* and its metabolites in the host. Extracellular vesicles (EVs) are spherical with a lipid bilayer secreted by all living cells in different ways. EVs contain various types of molecules, such as proteins, RNA, DNA, and lipids, and are the major communication substances between microorganisms and hosts (20). Increasing evidence has demonstrated that EVs are recognized by host cells and can regulate signaling pathways as well as the physiological and pathological processes of hosts (21, 22). It has been reported that *Lactobacillus reuteri*-derived EVs reduce TNF-α, IL-1β, and IL-6 expression by inhibiting the activity of the NF-κB pathway, thus inhibiting the inflammatory reaction induced by lipopolysaccharide (LPS) (23). It has also been reported that EVs of *Streptococcus pneumoniae* can activate autophagy and destroy alveolar epithelial barrier function by reducing occludin expression in alveolar epithelial cell A549 (24). *Bacteroides acidophilus* and its derived EVs can alleviate dextran sulfate sodium (DSS)-induced enteritis by restoring the diversity of intestinal microbes and increasing the expression of the intestinal tight junction proteins, occludin, and ZO-1 (25).

Toll-like receptors (TLRs) are essential pathogen-specific recognition sensors in the innate immune system (26–29). TLR4 is the most important receptor that mediates the LPS response and is extensively distributed in the macrophages, lungs, lymphocytes, spleen, and other tissues (30). LPS binds to TLR4, which transmits signals to cells, and the intracellular TLR region binds to the carboxyl end of MyD88 (31). Through a series of signal transduction, the NF-κB inhibitor is phosphorylated and degraded. NF-κB and its inhibitor protein are dissociated and transferred to the nucleus, activating cytokines IL-6, IL-1β, TNF-α, and so on (32). A study demonstrates that the essential oil from *Cinnamomum cassia* can improve LPS-induced ALI by inhibiting TLR4/MyD88/NF-κB signaling pathway (33). Another study reported that total polyphenols from *Nymphaea candida* can prevent LPS-induced ALI by regulating intestinal microbe and inhibiting the TLR4/NF-κB signaling pathway (34).

This study aimed to investigate whether intranasal administration of *C. butyricum* and its EVs can prevent ALI. Herein, after the pretreatment of mice with *C. butyricum* and EVs for 7 days, ALI was induced using LPS to reveal the anti-inflammatory, anti-apoptotic, and mucosal barrier-protective effects of *C. butyricum* and EVs in ALI mice. Moreover, we revealed the effects of pretreatment with *C. butyricum* on the lung microbiota of mice with ALI. Thus, this study provides a theoretical basis for intranasal administration of probiotics for the treatment of lung diseases.

## MATERIALS AND METHODS

### Animal model and treatments

Female BALB/c mice (6–8 weeks old, 20–22 g) were purchased from Changsha Tianqin Biotechnology Co., Ltd. Mice were free to get food and water. They were fed adaptively for 1 week under the conditions of a standard temperature of 22 ℃, suitable humidity, and 12 h of light/dark circulation. BALB/c mice were randomly divided into three groups (*n* = 8): normal control (Ctrl), free access to food and water. Model group (LPS group): the LPS group was administered the same volume of normal saline for 7 days. One hour after the nasal drip on the last day, 5 mg/kg of LPS was injected into the trachea to make the model (35). Treatment group (CB group): the CB group was pretreated with 20 µL *C*. *butyricum* (1 × 10^8^ CFUs) by nasal drip for 7 days. One hour after the nasal drip on the last day, 5 mg/kg of LPS (Cat# L8880, Solarbio, Inc.) was injected into the trachea to create a model. Mice were euthanized 24 h post-LPS stimulation. To further determine the role of *C. butyricum* EVs (CEVs) in acute lung injury mice model, nasal CEV (50 µg/mL) was pretreated for 7 days.

### Culture of bacteria

*C. butyricum* (BNCC337239, BeNa, Henan, China) was cultured in reinforced clostridial medium (RCM) under anaerobic conditions at 37°C for 18–24 h (19). The bacterial concentration was computed as the number of bacterial CFUs on RCM agar plates.

### Isolation, identification, and labeling of EVs from *C. butyricum*

*C. butyricum* was cultured in RCM for 24 h. The culture solution was centrifuged at 15,000 × *g* and 4°C for 25 minutes. The supernatant was filtered with 0.45 µm polyvinylidene fluoride (PVDF) membrane on vacuum filtration equipment to remove larger polymer particles. It was then filtered with a 0.22-µm filter to remove residual bacteria. The filtered supernatant was concentrated using an Amicon ultrafiltration system. The concentrated liquid was transferred to a 50 mL centrifuge tube. The filter and membrane were washed with sterilized phosphate-buffered saline (PBS). The retained liquid was collected in a centrifuge tube. After swirling the concentrated solution for 20 minutes, it was centrifuged at 12,000 × *g* and 4°C for 20 minutes. The supernatant was filtered with a 0.22-µm filter membrane. The last concentrated solution was centrifuged with an ultracentrifuge at 4°C and 150,000 × *g* for 90 minutes. The precipitate was kept, and the supernatant was discarded. The precipitate was a bacterial extracellular vesicle that may contain small amounts of impurities. Bacterial EVs were purified by gradient density centrifugation, and 1 mL of OptiPrep solution (10%–50%, vol/vol) at different concentrations was added in turn from high to low concentrations to cover the bacterial extracellular vesicle samples. Centrifugation was done at 180,000 × *g* and 4°C for 16 h. Then, 1 mL of each layer was absorbed, and after dilution with PBS, the number of particles in each layer was determined using a nanoparticle tracking analyzer. The density layers with high extracellular vesicle content of bacteria were merged and diluted with PBS. They were centrifuged at 4°C and 150,000 × *g* for 2 h, and the supernatant was discarded. It was then washed with PBS thrice to completely remove the OptiPrep. The precipitate was resuspended in PBS. A bicinchoninic acid (BCA) protein detection kit (Cat# PC0020, Solarbio, Inc.) was used to detect the protein content of the EVs. EVs were stored in a refrigerator at −80°C (36, 37).

### TEM

The purified exocrine sample suspension was dropped onto a copper mesh support membrane. It was allowed to stand at room temperature for 1 minute, and then the excess liquid was absorbed. The EV samples were dried, fixed with glutaraldehyde, and washed thrice with PBS. The samples were then dehydrated with ethanol at different concentration gradients. They were air-dried for 5–10 minutes and observed under a transmission electron microscope (38).

### Western blotting analysis

Proteins were extracted from mouse lung tissue samples using radioimmunoprecipitation assay (Cat# R0010, Solarbio, Inc.) tissue lysate, which contained phenylmethanesulfonyl fluoride (Cat# P0100, Solarbio, Inc.) and a protease inhibitor cocktail (Cat# HY-K0022, Med Chem Express, Inc., China). Protein content was determined using a BCA protein detection kit (Cat# PC0020, Solarbio, Inc.). After electrophoresis and membrane transfer, the proteins were transferred to PVDF membranes and sealed with 5% skim milk powder for 90 minutes (Cat# P1622-1, Beijing Applygen Technologies, Inc., China). The membrane was incubated overnight at 4°C with the following primary antibodies, rabbit anti-ZO-1 (1:5,000, Cat# 21773-1-AP), rabbit anti-occludin (1:5,000, Cat# 27260-1-AP), mouse anti-B-cell lymphoma 2 (Bcl2, 1:10,000, Cat# 68103-1-Ig), mouse anti-Bcl-2-associated X protein (BAX, 1:10,000, Cat# 60267-1-Ig), rabbit anti-Caspase 3 (1:1,000, Cat# 19677-1-AP), mouse anti-cleaved-caspase 3 (1:5,000, Cat# 68773-1-Ig), mouse anti-TLR4 (1:4,000, Cat# 66350-1-Ig), rabbit anti-NF-κB (1:2,000, Cat# 10745-1-AP), rabbit anti-Phospo-NF-κB (1:2,000, Cat# 82335-1-RR), rabbit anti-MyD88 (1:2,000, Cat# 23230-1-AP), and mouse anti-glyceraldehyde-3-phosphate dehydrogenase (GAPDH, 1:5,000, Cat# 60004-1-Ig). The antibodies were obtained from Proteintech Group, Inc. The membrane was washed thrice with 1× Tris-buffered-saline-Tween-20 buffer, each time for 10 minutes. At room temperature, the PVDF membrane was incubated with horseradish peroxidase (1:5,000, Cat# bs-0295G-HRP, Bioss, Inc.) and a goat anti-mouse secondary antibody (Cat# bs-0296G-HRP, Bioss, Inc.) for 1 h. The cells were exposed to a hypersensitive enhanced chemiluminescence reagent (Cat# PA112, Tiangen Biotech Co. Ltd., China) (39).

### H and E, immunofluorescence, and TUNEL staining

Lung tissues were fixed with 4% paraformaldehyde solution, embedded in paraffin, and made transparent with xylene. They were washed with distilled water, stained with hematoxylin and eosin (H and E), dehydrated with ethanol, and sealed with neutral gum. The images were captured using an inverted microscope. To detect apoptosis in the lung tissue of mice, staining was conducted using a transferase-mediated dUTP nick-end labeling (TUNEL) staining kit (Cat# G1504-50T, Servicebio, China). To observe the morphological changes and protein expression in the lungs of mice, immunofluorescence tests for ZO-1 and occludin were conducted (ZO-1, 1:500, Cat# GB111402-100, Servicebio, China and occludin, 1:750, Cat# GB111401-100, Servicebio, China). The images were obtained using a fluorescence microscope (25, 40).

### ELISA

Mouse blood was centrifuged at 1,000 × *g* and 4℃ for 15 minutes to obtain supernatant. Bronchoalveolar lavage fluid (BALF) was collected and centrifuged at 400 × *g* at 4℃ for 10 minutes (25). The concentrations of cytokines IL-1β (Cat# RK00006, Abclonal, China), IL-6 (Cat# RK0008, Abclonal, China), and TNF-α (Cat# RK00027, Abclonal, China) in serum and bronchoalveolar lavage fluid sample were detected in strict accordance with the instructions of enzyme-linked immunosorbent assay (ELISA) kit. Through the OD value of the sample, the concentration value of inflammatory factors in the detected sample can be obtained from the standard curve. When calculating the concentration of diluted samples, it needs to be multiplied by the dilution multiple.

### Quantitative reverse transcription polymerase chain reaction analysis

Total RNA was extracted from the lung tissue using TRIzol Substitute (Cat# SM129-02, Seven, China). RNA purity and concentration were determined using an ultraviolet spectrophotometer. Using the All-in-one First Strand cDNA Synthesis Kit (Cat# SM134-02, Seven, China), 20 µL of reaction system was synthesized according to the instructions of the kit. cDNA was stored at −20°C for later use. The prepared cDNA was amplified by polymerase chain reaction (PCR). The amplification primers were synthesized by Beijing Qingke Biotechnology Co., Ltd., and primer sequences were as follows: IL-6, 5′-CCAAGAGGTGAGTGCTTCCC-3′ (forward) and 5′-CTGTTGTTCAGACTCTCTCCCT-3′ (reverse). IL-1β, 5′-GCAACTGTTCCTGAACTCAACT-3′ (forward) and 5′-ATCTTTTGGGGTCCGTCAACT-3′ (reverse). TNF-α, 5′-CCCTCACACTCAGATCATCTTCT-3′ (forward) and 5′-GCTACGACGTGGGCTACAG-3′ (reverse). GAPDH, 5′-AGTATGACTCCACTCACGGC-3′ (forward) and 5′-CACCAGTAGACTCCACGACA-3′ (reverse) (41).

### DNA extraction and 16S rRNA gene sequencing

Lung tissues from mice in each group were collected for 16S rRNA gene sequencing. Total microbial genomic DNA (Tiangen Biotechnology Co., Ltd.) was extracted from mouse lung tissue according to the manufacturer’s instructions for the genomic DNA kit. The DNA samples were sent to Shanghai Biotechnology Co., Ltd. (Shanghai, China). The composition and relative abundance of the lung microbiota were analyzed using Qualcomm 16S rRNA gene sequencing technology. The primer corresponds to region V4, and the primer sequence (515F, 5′-AYTGGYDTAAGNG-3′, 806R, 5′-TACNVGGTTACTAATCC-3′) and the PCR products were paired and sequenced by Illumina HiSeq 2000 platform (Illumina, Inc., San Diego, CA, USA). Tags with more than 97% similarity were grouped into the operational classification unit (OTU). Using high-quality statistical data on sequence length distribution and a reference sequence database, the species were classified according to their phylum, class, order, family, genus, and species (42).

### Statistical analysis

GraphPad Prism 8.0 software was used for statistical analysis, and the data were expressed as mean ± standard deviation. One-way ANOVA was used for comparison among groups, and there was a significant statistical difference when *P* < 0.05 was used.

## RESULTS

### *C. butyricum* alleviates LPS-induced ALI

**Fig 1:**
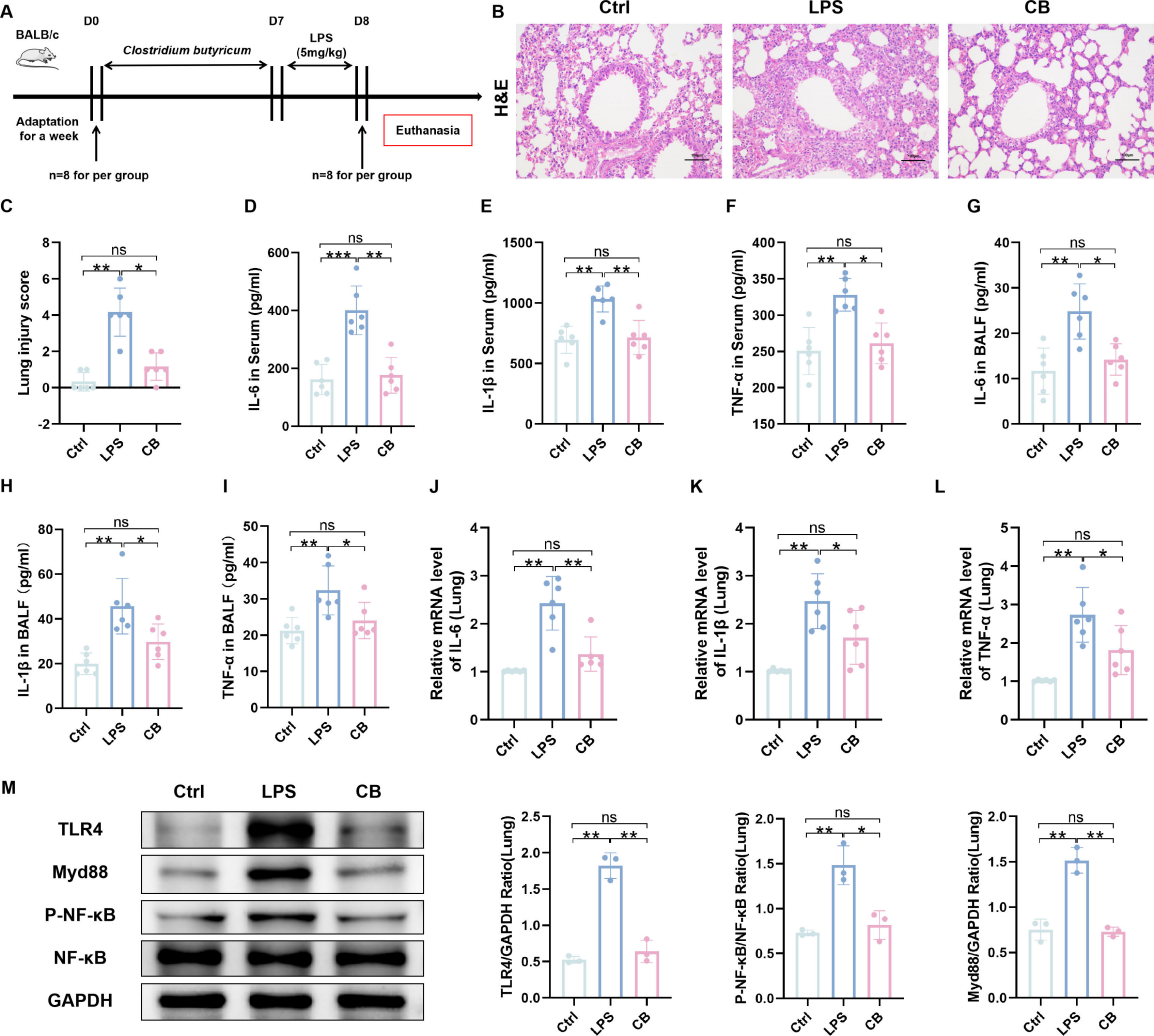
*Clostridium butyricum* attenuates LPS-induced ALI in mice. (**A**) Experimental design. (**B**) H and E staining of lung tissue sections. (**C**) Pathological scores of lung tissue sections. (**D–F**) Serum levels of IL-6, IL-1β, and TNF-α. (**G–I**) Levels of IL-6, IL-1β, and TNF-α in BALF. (**J–L**) Relative mRNA expression levels of IL-6, IL-1β, and TNF-α in lung tissue. (**M**) Expression of key proteins of TLR4/MyD88 signaling pathway in lung tissue samples. Ctrl, control group. LPS, LPS group. CB, ALI mice pretreated with nasal drops of *C. butyricum*. Scale bar, 100 µm. The data are expressed as mean ± SD. ns, *P* > 0.05; *P < 0.05; **P < 0.01; and ***P < 0.001.

To observe the alleviating effect of *C. butyricum* on ALI in mice, we pretreated mice with *C. butyricum* intranasally for 7 days and established an ALI mouse model by intratracheal injection of LPS (Fig. 1A). Histological analysis revealed that, compared with the LPS group, the CB group demonstrated significantly reduced inflammatory cell infiltration, capillary congestion in the alveolar wall, and a thinner pulmonary septum (Fig. 1B and C). To evaluate the effect of *C. butyricum* on the systemic inflammatory response in ALI mice, we measured pro-inflammatory cytokines in the serum and bronchoalveolar lavage fluid of mice. As depicted in Fig. 1D through I, LPS treatment remarkably increased the levels of proinflammatory cytokines IL-6, IL-1β, and TNF-α in serum and BALF, while intranasal administration of *C. butyricum* greatly reversed this trend. Additionally, we detected the expression of pro-inflammatory cytokines IL-6, IL-1β, and TNF-α in lung tissue at the RNA level. The results demonstrated that LPS treatment increased the levels of IL-6, IL-1β, and TNF-α, while *C. butyricum* pre-treatment decreased the levels of these pro-inflammatory cytokines in lung tissue (Fig. 1J through L). To explore the role of the TLR4/MyD88 signaling pathway in ALI prevention by *C. butyricum*, we detected the expression of key proteins in this signaling pathway, such as TLR4, MyD88, and NF-κB. LPS treatment led to the significant upregulation of TLR4 and MyD88. It promoted the phosphorylation of NF-κB, while the pretreatment of *C. butyricum* reversed this result (Fig. 1M). These results indicate that *C. butyricum* alleviates LPS-induced ALI by inhibiting the TLR4/MyD88 signaling pathway and reducing the release of inflammatory factors.

### *C. butyricum* increased the expression of lung barrier proteins and inhibited the apoptosis of LPS-induced ALI mice

**Fig 2:**
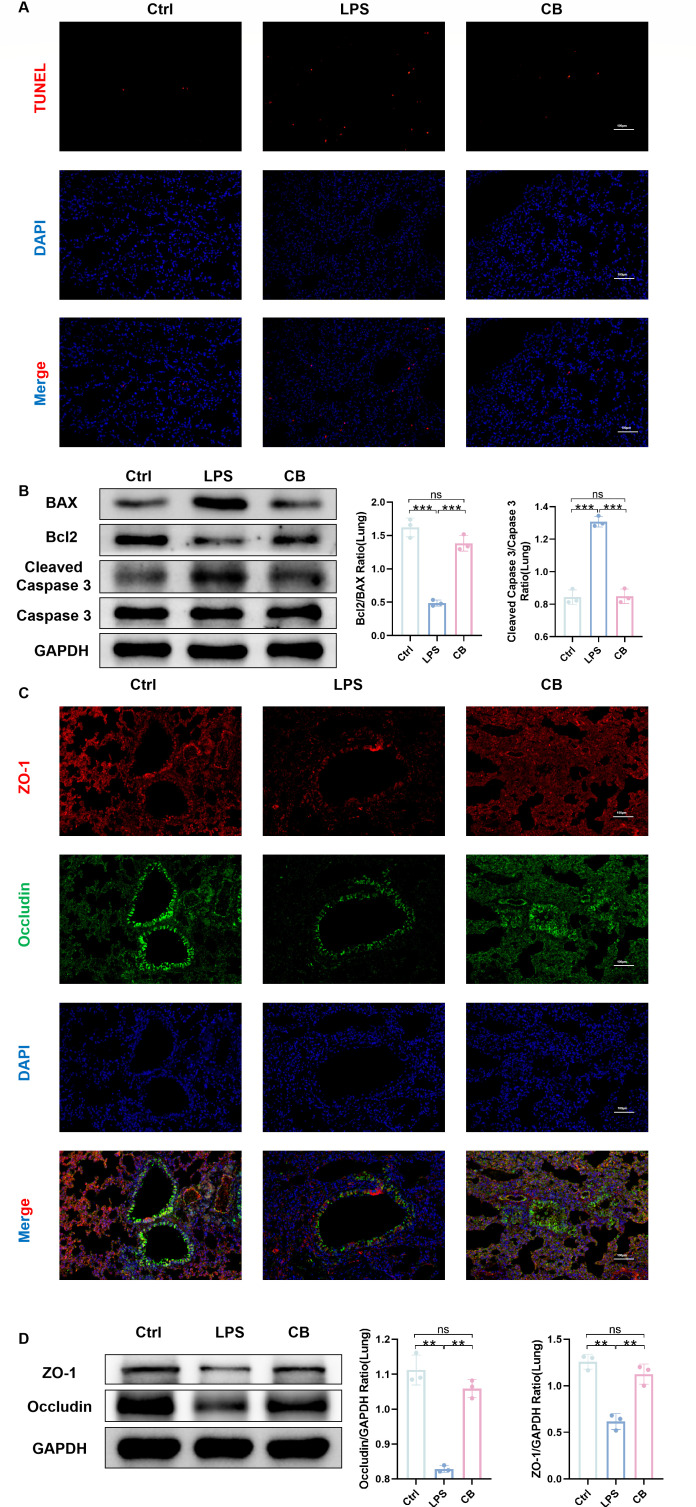
*C. butyricum* increases the expression of lung barrier protein and inhibits apoptosis in ALI mice. (**A**) Representative fluorescence pictures of TUNEL staining in lung sections. (**B**) Expression of apoptosis-related proteins (BAX, Bcl2, Caspase 3, and Cleaved Caspase 3) in the lung tissue of mice. (**C**) The expression of tight junction proteins (ZO-1 and occludin) in lung tissue sections is detected by immunofluorescence. (**D**) Expression of tight junction protein (ZO-1 and occludin) in mouse lung tissue. Ctrl, control group. LPS, LPS group. CB, ALI mice pretreated with nasal drops of *C. butyricum*. Scale bar, 100 µm. The data are expressed as mean ± SD. ns, *P* > 0.05; *P < 0.05; **P < 0.01; and ***P < 0.001.

We investigated the effects of *C. butyricum* on LPS-induced apoptosis in mice with ALI. TUNEL analysis demonstrated that LPS induction significantly increased apoptosis in lung tissue, whereas intranasal administration of *C. butyricum* reversed this trend (Fig. 2A). We detected the expression of apoptosis-related proteins, such as BAX, Bcl-2, Caspase-3, and Cleaved Caspase-3 proteins. Compared with the LPS group, the *C. butyricum* pre-treatment group demonstrated obviously downregulated expression of BAX and Cleaved Caspase-3 proteins and upregulated expression of Bcl-2 protein (Fig. 2B). Additionally, we determined the protective effect of *C. butyricum* on the lung barrier. The expression of barrier proteins (ZO-1 and occludin) demonstrated that intranasal administration of *C. butyricum* observably reversed the LPS-induced reduction in lung barrier proteins (Fig. 2C and D). These results indicate that the intranasal administration of *C. butyricum* can prevent LPS-induced apoptosis of lung tissue cells and protect lung mucosal barrier function.

### *C. butyricum*-induced EVs alleviate LPS-induced ALI

**Fig 3:**
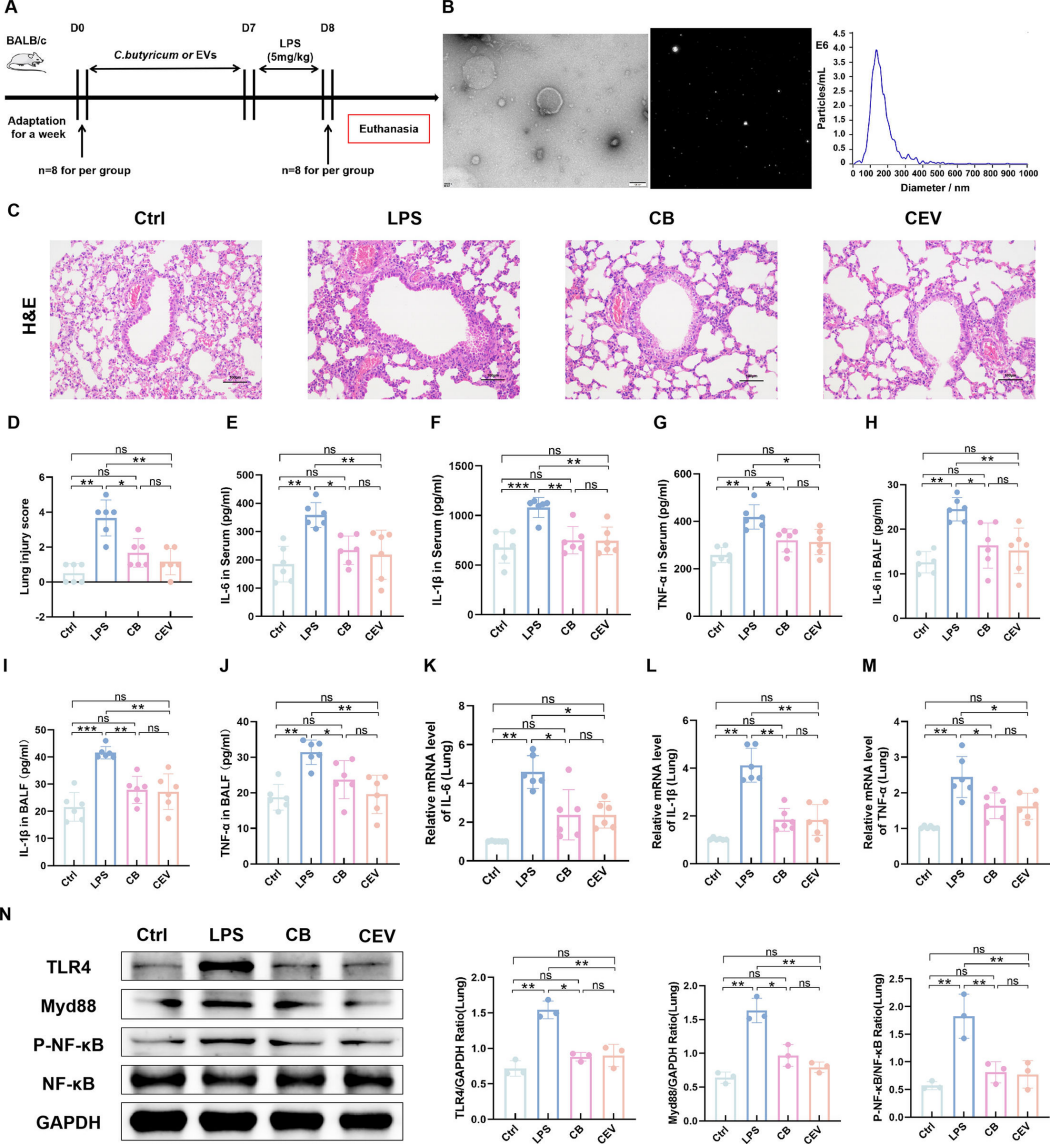
EVs derived from *C. butyricum* alleviate LPS-induced ALI. (**A**) Experimental design. (**B**) TEM and NTA of EVs derived from *C. butyricum*. Scale bar, 100 nm. (**C**) H and E staining of lung tissue sections. (**D**) Pathological score of lung tissue sections. (**E–G**) Serum levels of IL-6, IL-1β, and TNF-α (**H–J**) Levels of IL-6, IL-1β, and TNF-α in BALF. (**K–M**) Relative mRNA expression levels of IL-6, IL-1β, and TNF-α in lung tissue. (**N**) Expression of key proteins in TLR4/MyD88 signaling pathway in mouse lung tissue samples. Ctrl, control group. LPS, LPS group. CB, ALI mice pretreated with nasal drops of *C. butyricum*. CEV, ALI mice pretreated with nasal drops of *C. butyricum* EVs. Scale bar, 100 µm. The data are expressed as mean ± SD. ns, *P* > 0.05; *P < 0.05; **P < 0.01; ***P < 0.001.

We investigated whether EVs derived from *C. butyricum* could alleviate LPS-induced ALI. We isolated and purified EVs from *C. butyricum* and pretreated ALI mice for 7 days via intranasal administration (Fig. 3A). We obtained the morphology and diameter of CEV using transmission electron microscopy (TEM) and nanoparticle tracking analysis (NTA). TEM demonstrated that EVs derived from *C. butyricum* have a saucer-like vesicle morphology. NTA demonstrated that the diameter of the EVs was approximately 135.1 nm (Fig. 3B). We used H and E staining to evaluate the effect of LPS on lung histopathology in mice with ALI. The lung tissues of ALI mice in the LPS group demonstrated inflammatory cell infiltration, alveolar capillary congestion, pulmonary septum thickening, and higher lung histological scores. However, in the CEV group, there were no inflammatory cells in the lung tissue. There was no congestion in the alveoli, indicating that CEV can alleviate the pathological damage to lung tissue (Fig. 3C and D). Additionally, to explore the effect of CEV on the systemic response of ALI mice, we measured the expression of pro-inflammatory cytokines in the serum and BALF of the mice. Compared to the LPS group, intranasal administration of CEV remarkably decreased the concentrations of pro-inflammatory cytokines IL-6, IL-1β, and TNF-α in the serum (Fig. 3E through G), BALF (Fig. 3H through J), and lung tissue (Fig. 3K through M). Similarly, we detected the expression of key proteins in the lung tissue to explore the regulatory effect of CEV on the TLR4/MyD88 signaling pathway in LPS-induced ALI mice. The results demonstrated that the pre-treatment of intranasal administration of CEV significantly reduced the protein expression of TLR4 and MyD88 and inhibited the phosphorylation of NF-κB (Fig. 3N). This indicated that CEV inhibited the TLR4/MyD88 signaling pathway.

### CEV increased the expression of lung barrier proteins and inhibited the apoptosis of LPS-induced ALI mice

**Fig 4:**
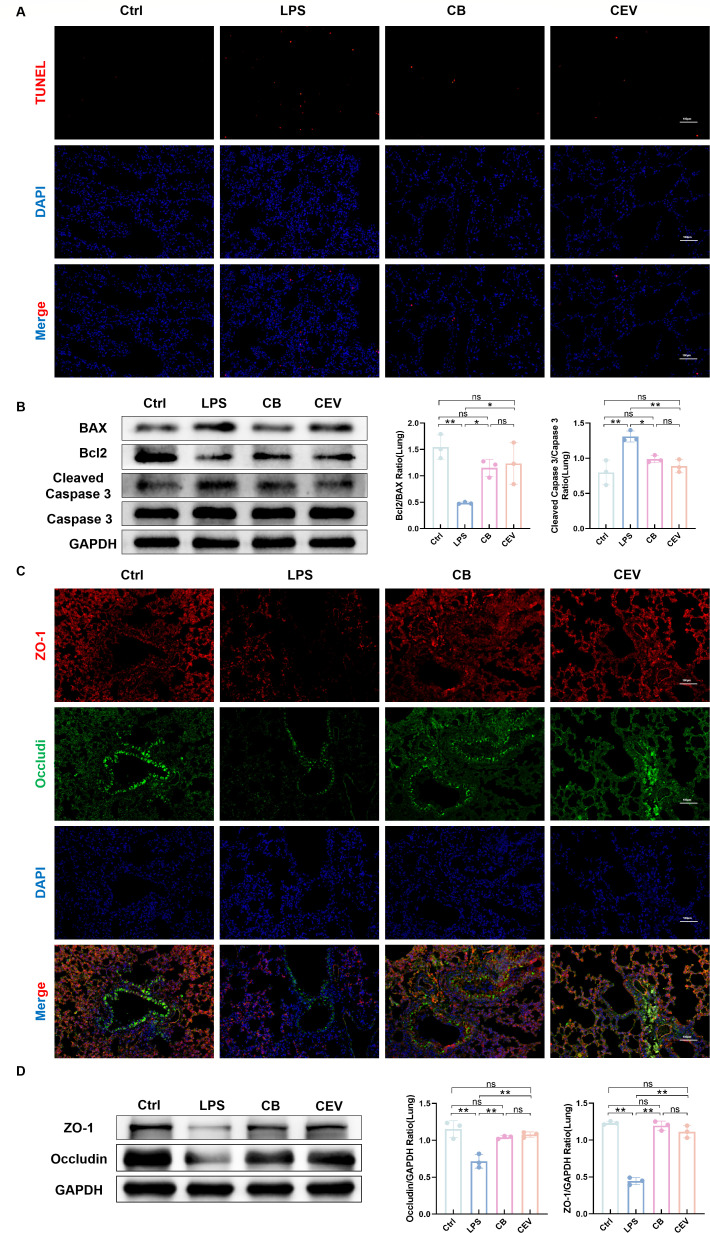
CEV increases the expression of lung mucosal barrier protein and inhibits the apoptosis of ALI mice. (**A**) Representative fluorescence pictures of TUNEL staining in lung sections. (**B**) Expression of apoptosis-related proteins (BAX, Bcl2, Caspase 3, and Cleaved Caspase 3) in the lung tissue of mice. (**C**) Immunofluorescence representative images of tight junction proteins (ZO-1 and occludin) on lung tissue sections. (**D**) Expression of tight junction proteins (ZO-1 and occludin) in the lung tissue of mice. Ctrl, control group. LPS, LPS group. CB, ALI mice treated with nasal drops of *C. butyricum*. CEV, ALI mice treated with nasal drops of *C. butyricum* EVs. Scale bar, 100 µm. The data are expressed as mean ± SD. ns, *P* > 0.05; *P < 0.05; **P < 0.01; and ***P < 0.001.

Exploring the effect of CEV on LPS-induced apoptosis in ALI mice TUNEL analysis demonstrated that intranasal administration of CEV considerably reduced apoptosis in the lung tissue compared to that in the LPS group (Fig. 4A). We measured the expression of the apoptosis-related proteins BAX, Bcl2, Caspase 3, and Cleaved Caspase 3. The results demonstrated that the LPS group showed significantly increased expression of BAX and Cleaved Caspase 3 proteins in the lung tissue and decreased expression of Bcl2 protein. Supplementation with CEV inhibited lung cell apoptosis (Fig. 4B). Tight junction proteins play critical roles in controlling lung permeability. To explore the effects of CEV on the lung barrier, we measured the expression of lung tight junction proteins (ZO-1 and occludin). The results demonstrated that nasal administration of CEV observably increased the expression of ZO-1 and occludin in the lung tissue compared with the LPS group (Fig. 4C and D). These results indicate that the intranasal administration of CEV can inhibit lung tissue apoptosis in ALI mice and protect the function of the lung mucosal barrier.

### *C. butyricum* regulated the composition of pulmonary microbiota

**Fig 5:**
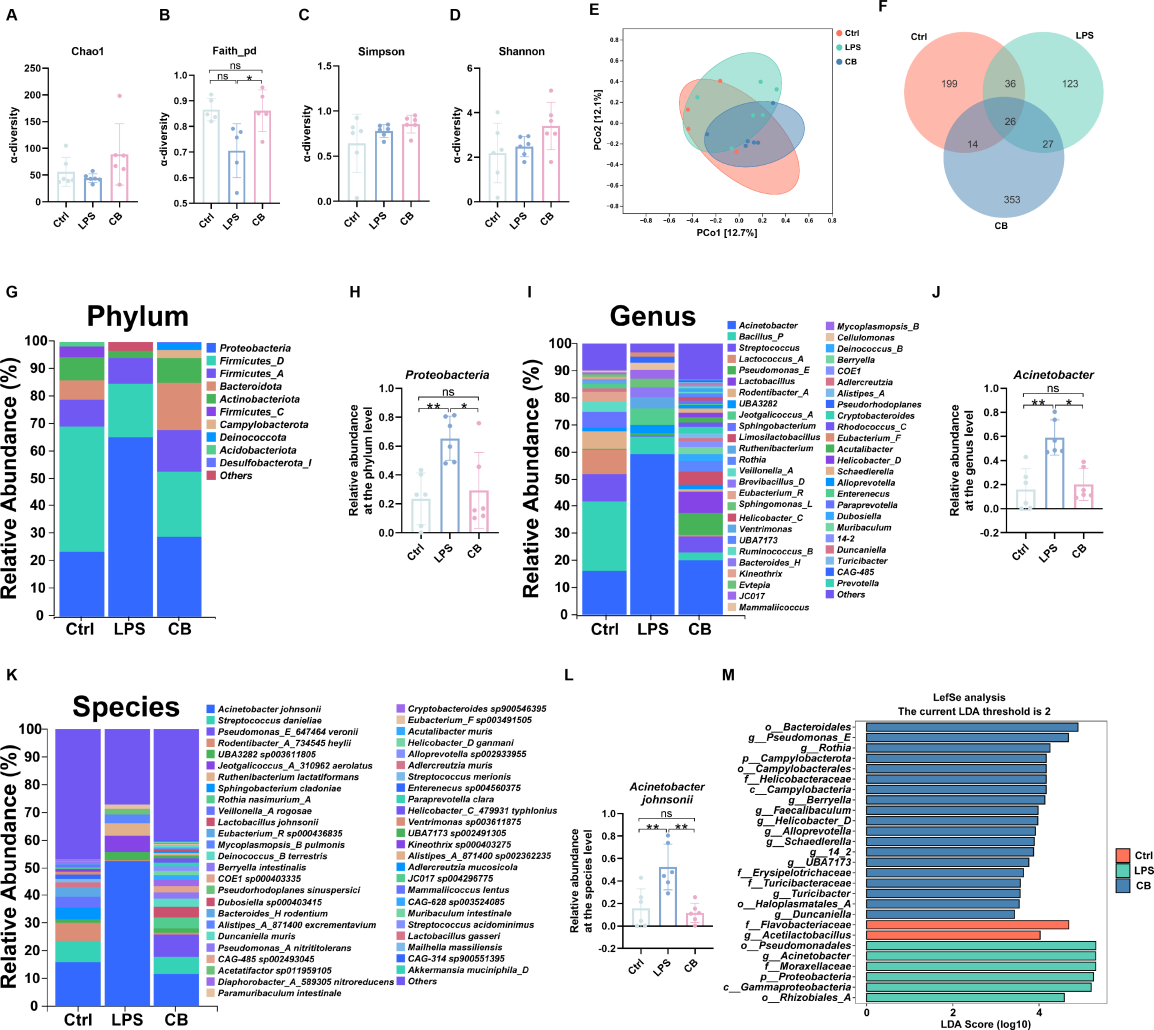
*C. butyricum* changes the composition of pulmonary microbiota in ALI mice induced by LPS. (**A**) Chao1 index. (**B**) Faith_pd index. (**C**) Simpson index. (**D**) Shannon index. (**E**) PCoA analysis of β diversity. (**F**) Venn diagram. (**G, I, and K**) According to the sequencing of 16S rRNA gene, the relative abundance of pulmonary microbiota was identified at the level of (**G**) phylum, (**I**) genus, and (**K**) species. (**H**) *Proteobacteria.* (**J**) *Acinetobacter.* (**L**) *Acinetobacter johnsonii.* (**M**) LefSe analysis has identified microorganisms with significantly different abundances among Ctrl group, LPS group, and CB group (LDA > 2). Ctrl, control group. LPS, LPS group. CB, ALI mice pretreated with nasal drops of *C. butyricum*. Scale bar, 100 µm. The data are expressed as mean ± SD. ns, *P* > 0.05; *P < 0.05; **P < 0.01; and ***P < 0.001.

We further explored the effect of *C. butyricum* on the composition of the pulmonary microbiota in LPS-treated mice using 16S rRNA gene sequencing. The α-diversity analysis demonstrated that LPS treatment reduced the α-diversity of pulmonary microbiota in mice. *C. butyricum* treatment reversed the decrease of α-diversity of pulmonary microbiota, including Chao1 (Fig. 5A), Faith_pd (Fig. 5B), Simpson (Fig. 5C), and Shannon (Fig. 5D). Principal coordinate analysis (PCoA) demonstrated that LPS caused the pulmonary microbiota to move farther away from the Ctrl group, whereas *C. butyricum* pretreatment group approached the Ctrl group (Fig. 5E). Additionally, the Venn diagram (Fig. 5F) showed 275, 212, and 420 OTUs in the control, LPS, and CB groups, respectively. The proportions of common OTUs in each group were 9.45% (26/275), 12.26% (26/212), and 6.19% (26/420), respectively. We discussed the composition of the lung microbiota and bacteria related to lung diseases at the phylum, genus, and species levels. *Proteobacteria*, *Firmicutes_D*, *Firmicutes_A,* and *Bacteroidota* were the major phylum in the pulmonary microbiota (Fig. 5G). The results demonstrated that the relative abundance of *Proteobacteria* in LPS-induced ALI mice increased significantly, whereas the use of *C. butyricum* reversed the relative abundance of *Proteobacteria* (Fig. 5H). At the genus level, the pulmonary microbiota predominantly comprised *Acinetobacter*, *Bacillus_P,* and *Streptococcus* (Fig. 5I). Further analysis demonstrated that the relative abundance of *Acinetobacter* in LPS-induced ALI mice increased dramatically, whereas the administration of *C. butyricum* sharply decreased the relative abundance of *Acinetobacter* in ALI mice (Fig. 5J). The results of the species-level analysis demonstrated that the relative abundance of *Acinetobacter johnsonii* in LPS-induced ALI mice increased remarkably, whereas the use of *C. butyricum* reversed this effect (Fig. 5K and L). Subsequently, linear discriminant analysis effect size (LefSe) analysis (Fig. 5M) demonstrated that the dominant bacteria in the CB group were *Bacteroidales* (order), *Pseudomonas _ E* (genus), and *Rothia* (genus). The dominant bacterial populations in the control group were *Flavobacteriaceae* (family) and *Acetilactobacillus* (genus). The dominant bacteria in the LPS group were *Pseudomonadales* (order), *Acinetobacter* (genus), and *Moraxellaceae* (family). These results indicated that LPS induction markedly altered the composition of the lung microbiota, whereas pretreatment with *C. butyricum* significantly restored the balance of the lung microbiota in mice.

## DISCUSSION

**Fig 6:**
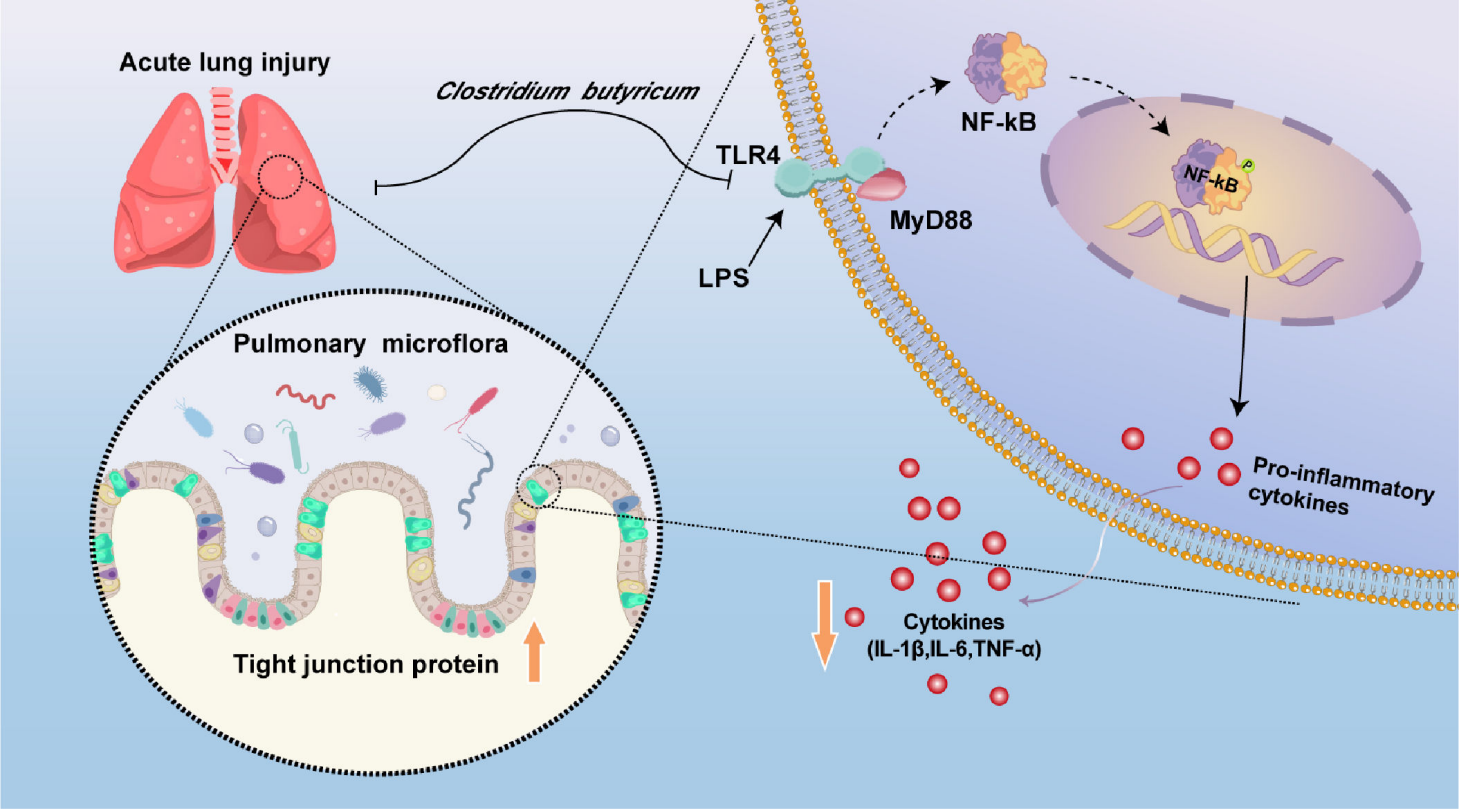
A schematic diagram summarizing the main findings of this study.

ALI is characterized by an ecological imbalance in inflammatory cytokines and destruction of the lung epithelial barrier, with high morbidity and mortality (43, 44). In the normal composition of the pulmonary microbiota, *Bacteroides* and *Firmicutes* are the most common genera at the phylum level, and *Streptococcus*, *Prevotella*, and *Veillonella* are the most common genera (45). The incidence of ALI is closely related to pulmonary microbiota disorders and adjusting the composition of pulmonary microbiota can ameliorate ALI (6). Here, we explored whether *C. butyricum* and its metabolites have therapeutic effects on ALI. In this study, we explored whether *C. butyricum* could alleviate the symptoms of LPS-induced ALI in mice by inhibiting the TLR4/MyD88 signaling pathway, reducing inflammatory reactions, inhibiting apoptosis, and repairing the lung mucosal barrier. Subsequently, we demonstrated that EVs derived from *C. butyricum* alleviated the symptoms of ALI in mice by inhibiting inflammatory responses, reducing cell apoptosis, and protecting the lung barrier. We verified that *C. butyricum* could regulate LPS-induced pulmonary microbiota imbalance (Fig. 6).

*C. butyricum* is an anaerobic bacterium that produces butyric acid and spores and is extensively found in humans, rats, chickens, and pigs (46–48). Previous studies have verified that *C. butyricum* regulates the microbial composition, improves barrier function, and inhibits inflammation. A previous study found that *C. butyricum* could treat DSS-induced colitis by lowering the disease activity index score, reducing the expression of proinflammatory cytokines, and improving intestinal barrier function (37). Additionally, *C. butyricum* can inhibit inflammatory reactions in mice with primary nephrotic syndrome by regulating the balance of Th17/Tregs (49). Herein, we found that *C. butyricum* could alleviate LPS-induced lung inflammation based on histological scores and the detection of inflammatory cytokines in the serum and BALF. Another study found that *Lactobacillus plantarum* improved DSS-induced ulcerative colitis by reducing inflammatory cytokines, regulating microbial composition, and increasing barrier proteins (50). *Lactobacillus reuteri* can improve inflammation induced by *Escherichia coli K88* by inhibiting the myosin light chain kinase signaling pathway, reducing inflammatory cytokines, and enhancing the intestinal epithelial barrier (51). Moreover, *Bacillus* spores can treat children infected with influenza virus by reducing the viral load (52).

As a critical transmembrane recognition receptor of the innate immune system, TLR4 is involved in the occurrence of pneumonia and other inflammatory diseases, and NF-κB and MyD88 are its downstream molecules (53). It has been reported that LPS stimulates the body, and TLR4 is activated. This can induce NF-κB to be activated, and NF-κB is ectopic to the nucleus. This causes the release of pro-inflammatory cytokines, leading to lung injury (54). Previous studies have reported that LPS-induced ALI can be improved by inhibiting the TLR4/MyD88 signaling pathway (55, 56). It has been reported that Yupingfengsan can treat ALI by inhibiting TLR4/MyD88 signal pathway and reducing the expression of pro-inflammatory cytokines IL-1β, IL-6, and TNF-α (57). Herein, *C. butyricum* reduces the release of inflammatory factors and improves LPS-induced ALI by inhibiting the TLR4/MyD88 signaling pathway.

There are epithelial cell layers on the surface of the airway and alveoli, which form a defense barrier to prevent the invasion of pathogenic microorganisms and are important protective barriers for the lungs (58). The barrier function of lung epithelial cells is mediated by tight junction proteins. It regulates diverse intracellular signals and is crucial for material exchange between lung cell environments (59). When inflammatory reactions occur in the lungs, a large number of proinflammatory factors are released. These induce cell apoptosis and lead to alveolar-capillary barrier damage, thus increasing vascular permeability and leading to pulmonary edema and alveolar gas dysfunction (60). Inflammatory reactions have been found to lead to apoptosis, damaged tight junction proteins, destruction of lung epithelial barrier function, increased permeability of water and proteins, and entry of infectious pathogens and exogenous toxins into the circulatory system, leading to acute lung injury (61). ALI can be treated by increasing the expression of lung tight junction proteins ZO-1 and occludin and maintaining the integrity of the endothelial cell barrier (62). Quercetin alleviates LPS-induced lung injury by upregulating the expression of tight junction protein (63). Herein, *C. butyricum* alleviates LPS-induced damage to lung barrier function. It is reported that estrogen-related receptor alpha can prevent ALI in rats by upregulating BCL2 protein and downregulating BAX protein (64). Shufeng Jiedu capsules inhibit apoptosis by inhibiting NF-κB and treating ALI induced by LPS (65). Herein, LPS-induced ALI led to the apoptosis of lung tissue cells, whereas *C. butyricum* reduced this phenomenon.

Bacterial EVs play an important role in the communication between bacteria and host cells (66). Bacterial EVs are spherical bilayer protein lipids that are rich in bioactive proteins, lipids, and nucleic acids and regulate the function of host and recipient cells (67). To explore the interactions between *C. butyricum* and its host, we studied the role of EVs derived from *C. butyricum* in preventing ALI. EVs derived from *C. butyricum* improved the symptoms of ALI, including the histological score and serum and BALF proinflammatory factor expression. A previous study found that EVs derived from *Lactobacillus reuteri* maintain intestinal homeostasis and alleviate LPS-induced inflammation by reducing the release of proinflammatory factors (23). EVs of *C. butyricum* can reduce colonic histological score, inflammation, and intestinal barrier function to treat DSS-induced ulcerative colitis (37). EVs derived from the periodontopathogen *Aggregatibacter actinomycetemcomitans* can alleviate neurological diseases by downregulating the TLR4/MyD88 signaling pathway and reducing the expression of pro-inflammatory cytokines (68). Consistent with the above results, EVs from *C. butyricum* improve LPS-induced ALI by inhibiting the TLR4/MyD88 signaling pathway (69). Previous studies demonstrate an important relationship between inflammatory responses and mucosal barrier function (70–72). Herein, EVs of *C. butyricum* enhance the lung epithelial barrier function by regulating the expression of the tight junction proteins occludin and ZO-1. This stabilizes the distribution of lung barrier proteins and reduces the apoptosis of lung tissue cells. EVs derived from *Salmonella typhimurium* can alleviate colorectal cancer by promoting apoptosis and reducing tumor invasion (73). The EVs of *Streptococcus pneumoniae* destroy the protein expression of occludin in mouse lung tissue and A549 cells and the function of the lung epithelial barrier, resulting in serious lung injury (24). Herein, *C. butyricum* and its derived EVs reduced inflammatory reactions and apoptosis, repaired the mucosal barrier, and improved ALI by inhibiting the TLR4/MyD88 signaling pathway.

A study reported that *C. butyricum* can improve influenza virus pneumonia by up-regulating interferon-λ in lung epithelial cells (74). It has also been reported that the supernatant of *C. butyricum* has anti-biofilm activity and inhibits the proliferation of *Acinetobacter baumannii* by inhibiting biofilm development and metabolic activity of biofilm cells (75). Additionally, *C. butyricum* improves enteritis caused by *Salmonella* infection by reducing inflammation, increasing the expression of barrier proteins, and altering the composition of intestinal microorganisms (17). To explore the regulatory effect of *C. butyricum* on the pulmonary microbiota, we use 16S rRNA sequencing to analyze the changes in the pulmonary microbiota in each group of mice. Previous studies have demonstrated that LPS stimulation leads to a decrease in microbial diversity (76, 77). Herein, LPS induction significantly reduced the diversity of the pulmonary microbiota in mice, whereas supplementation with *C. butyricum* reversed this result. *Proteobacteria* is currently the largest phylum in the field of bacteria and has the characteristics of Gram-negative staining, including several known pathogens. The outer membrane of bacteria contains LPS. Evidence demonstrates that *Proteobacteria* are a possible microbial group involved in the disease (78). Herein, the relative abundance of *Proteobacteria* in the pulmonary microbiota of mice with LPS-induced ALI significantly increased. A previous study demonstrated that the relative abundance of *Acinetobacter* increased in mice with LPS-induced ALI (79). *Acinetobacter* has been proven to be easy to cause lung infection (80, 81). Additionally, a study demonstrated that *Acinetobacter* is an important risk factor for ALI induced by sepsis (82). *Acinetobacter* is a typical hospital pathogen that is prone to infection and death and has a high resistance rate (83, 84). Herein, the relative abundance of *Acinetobacter* in the pulmonary microbiota of mice with LPS-induced ALI increased significantly, whereas that of *Acinetobacter* in the *C. butyricum* treatment group decreased. It has been reported that *Acinetobacter johnsonii* is an important bacterial community in the nasal cavity of patients with chronic rhinitis and olfactory dysfunction (85). Additionally, *Acinetobacter johnsonii* can cause meningitis in children (86). It has also been reported that the relative abundance of *Acinetobacter johnsonii* in the oral microbiota of patients with recurrent aphthous stomatitis is significantly increased (87). Moreover, *Acinetobacter johnsonii* is one of the most common *Acinetobacter* in hospitals (88). Herein, *C. butyricum* supplementation significantly reduced the relative abundance of *Acinetobacter johnsonii*. Intranasal administration of *C. butyricum* alleviates LPS-induced ALI by modulating the pulmonary microbiota and reducing the relative abundance of *Proteobacteria* at the phylum level, *Acinetobacter* at the genus level, and *Acinetobacter johnsonii* at the species level.

In conclusion, *C. butyricum* and its derived EVs can reduce inflammatory reactions, inhibit cell apoptosis, reduce mucosal injury in the lung tissue, promote the repair of the mucosal barrier, and alleviate LPS-induced ALI. Additionally, *C. butyricum* can regulate the diversity of the pulmonary microbiota and restore the balance of pulmonary microbiota in mice. Herein, we clarified the effect of *C. butyricum* and its derived EVs on LPS-induced ALI and discussed the regulatory effect of *C. butyricum* on the pulmonary microbiota. However, how EVs communicate with the host and participate in host regulation may be the key to the treatment of ALI using *C. butyricum*, and further exploration is required. The results of this study provide a theoretical basis for the preclinical application of this new generation of probiotics (42, 89, 90).

## Data Availability

Data are provided within the manuscript or supplementary information files.
